# Microcystin Contamination in Irrigation Water and Health Risk

**DOI:** 10.3390/toxins16040196

**Published:** 2024-04-19

**Authors:** Mohammed Haida, Fatima El Khalloufi, Richard Mugani, Yasser Essadki, Alexandre Campos, Vitor Vasconcelos, Brahim Oudra

**Affiliations:** 1 Water, Biodiversity and Climate Change Laboratory, Faculty of Sciences Semlalia, Cadi Ayyad University, Marrakesh 40000, Morocco; mohammed.haida11@gmail.com (M.H.); richardmugani@gmail.com (R.M.); yasser.essadki@ced.uca.ma (Y.E.); oudra@uca.ac.ma (B.O.); 2 Natural Resources Engineering and Environmental Impacts Team, Multidisciplinary Research and Innovation Laboratory, Polydisciplinary Faculty of Khouribga, Sultan Moulay Slimane University of Beni Mellal, B.P, 45, Khouribga 25000, Morocco; elkhalloufi.f@gmail.com; 3 Interdisciplinary Centre of Marine and Environmental Research (CIIMAR), Terminal de Cruzeiros do Porto de Leixões, Av. General Norton de Matos, s/n, 4450-208 Porto, Portugal; acampos@ciimar.up.pt; 4 Department of Biology, Faculty of Sciences, University of Porto, Rua do Campo Alegre, 4169-007 Porto, Portugal

**Keywords:** microcystins, irrigation water, bioaccumulation, transfer, conjugation, health risk, phytotoxicity, biodegradation

## Abstract

Microcystins (MCs), natural hepatotoxic compounds produced by cyanobacteria, pose significant risks to water quality, ecosystem stability, and the well-being of animals, plants, and humans when present in elevated concentrations. The escalating contamination of irrigation water with MCs presents a growing threat to terrestrial plants. The customary practice of irrigating crops from local water sources, including lakes and ponds hosting cyanobacterial blooms, serves as a primary conduit for transferring these toxins. Due to their high chemical stability and low molecular weight, MCs have the potential to accumulate in various parts of plants, thereby increasing health hazards for consumers of agricultural products, which serve as the foundation of the Earth’s food chain. MCs can bioaccumulate, migrate, potentially biodegrade, and pose health hazards to humans within terrestrial food systems. This study highlights that MCs from irrigation water reservoirs can bioaccumulate and come into contact with plants, transferring into the food chain. Additionally, it investigates the natural mechanisms that organisms employ for conjugation and the microbial processes involved in MC degradation. To gain a comprehensive understanding of the role of MCs in the terrestrial food chain and to elucidate the specific health risks associated with consuming crops irrigated with water contaminated with these toxins, further research is necessary.

## 1. Introduction

Cyanobacteria proliferation has evolved as a direct result of climate change. This concerning trend is characterized by rising global temperatures and an increase in nutrient influx, mainly nitrogen and phosphorus, from sources such as agricultural runoff and wastewater treatment plants [1]. These cyanobacteria strains are potential toxins producers known as cyanotoxins. MCs are considered the most prevalent cyanotoxins in water bodies and have reached levels that exceed the limits set by the WHO (World Health Organization), posing a severe threat to worldwide public health [2,3].

In many underdeveloped and developing countries, water contaminated MCs is collected in lakes or artificial ponds and then used to irrigate all types of crops, including hydroponic crops, which are widespread due to the increase in human population and demand for agri-food products [4,5]. It should be noted that the presence of MCs in irrigation water is likely to induce a range of phytotoxic effects, including reduced seed germination, altered plant growth and development, reduced photosynthetic performance, and disruption of hormonal balance. These toxins also cause alterations in plant metabolism, such as increased lipid peroxidation, reduced protein content, and oxidative stress. As a result, yield losses attributable to MC exposure can range from 50% to 82%, depending on the specific crop and duration of exposure [6,7,8,9].

Irrigation water contaminated with MCs infiltrates and penetrates the root barriers of agricultural plants, and bioaccumulates in roots, stems, leaves, and edible crops, eventually entering the human body via consumption [10,11,12], with daily intakes above the recommended value of 0.04 μg of MC/kg of body weight/day [13]. 

The transfer of MCs through aquatic food chains has been the subject of several studies on a large number of organisms [14]. However, few studies have been conducted on MC transmission through terrestrial food chains. Little information is available about cases of MC transfer through the ingestion of contaminated food, involving bats, spiders, birds, and rats [15,16].

The bioaccumulation and transfer of MCs in the terrestrial food chain trigger adverse effects on animal and human health [17]. After entering the bloodstream, MCs cause severe damage to organs, such as the liver, kidneys, intestines, heart, brain, lungs, and gonads. MCs selectively attack these organs, causing long-term damage [18,19,20]. Although no case of human death has been recorded, exposure to MCs has been associated with the death of some animals [21,22,23,24,25].

Furthermore, MCs exhibit significant resistance to enzymatic hydrolysis [26]. Nevertheless, plant cells contaminated with MCs can undergo a natural detoxification process. In this process, MCs are converted into more hydrophilic compounds, which are then conjugated by enzymes such as glutathione S-transferase (GST) and glutathione (GSH). These compounds are then compartmentalized [27,28,29]. Studies have demonstrated that converted conjugates have substantially less toxicity than untransformed conjugates, even though the detoxification process does not completely remove MCs from plants [30].

Therefore, it is believed that the biological process of microorganism biodegradation in irrigation water offers an economical and effective means of purifying MC-contaminated water bodies [31]. First attempts have been made to isolate a strain of *Sphingomonas* from surface waters capable of using MC-LR as the only source of carbon and nitrogen for its growth. The results of these experiments showed that the degradation of 1 mg L^−1^ of MC–LR generally began 2 to 8 days after its addition to surface water samples [32]. The main objectives of this work were to determine the fate of MCs in irrigation water, investigate the phytotoxic effects of MCs on plants in hydroponic cultures, assess the bioaccumulation and transfer of MCs in a terrestrial food chain, determine the role of detoxification enzymes and bacterial strains capable of degrading MCs in the elimination of this toxin, and assess the risks associated with exposure to MCs for human and animal health. To achieve the objectives of this review, reference documents were collected using online databases such as Scopus, ScienceDirect, PubMed, and Web of Science, and journals have been carefully read and analyzed. Reference documents were collected using main keywords such as microcystins in irrigation water, bioaccumulation of microcystins in plants, transfer of microcystins in the food chain, conjugation of microcystins, health risk of microcystines, microcystine phytotoxicity, and microcystine biodegradation. The years of publication for reference elements were mainly between 1981 and 2023. More than 500 references were retrieved, and 147 articles were used as references in the manuscript.

## 2. Microcystins

**Figure 1:**
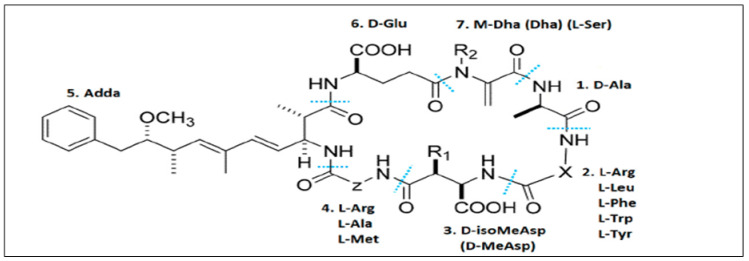
Structure of the hepatotoxic cyclic peptides, MCs. The numbers 1–7 represent seven amino acid residues. X and Z in positions two and four are highly variable L-amino acids [41].

The potential for cyanobacteria to produce harmful toxins to humans and animals makes their worldwide abundance a serious cause for concern [3]. Many cyanobacteria species produce secondary metabolites such as neurotoxins, dermatoxins, and hepatotoxins [33]. MCs have a cyclic heptapeptide structure: cyclo-(D-Ala1-X2-D-isoMeAsp3-Y4-Adda5-D-isoGlu6-Mdha7), where X and Y stand for the very L-amino acid pair [34] (Figure 1). Among these analogs, MC-LR (L: Leucine, R: Arginine) is the most hazardous and prevalent one, garnering significant interest in studies and reports [13].

The detrimental effects of MCs are believed to be caused by the Adda residue (3-amino-9-methoxy-2,6,8-trimethyl-10-phenyldeca-4,6-dienoic acid) [35]. Cyanobacteria capable of making MCs include mainly *Microcystis*, *Anabaena*, *Nostoc*, *Planktothrix*, and *Oscillatoria* [36,37,38,39]. The enormous impact of MCs is due to their capacity to cause acute hepatotoxicosis in both humans and animals, making them very harmful cyanotoxins with severe health and ecological concerns [40]. 

## 3. Emergence and Persistence of MCs in Irrigation Water

The synthesis and release of cyanotoxins are influenced by several factors. Physicochemical factors (phosphorus, nitrogen, iron, pH, temperature, and light) promote the growth of cyanobacteria and consequently the excessive release of MCs. Biological factors, where the production of MCs increases in the presence of competitors and predators, as well as genetic factors, because variation in the amount of MCs synthesized results from changes in the transcription of genes that encode this toxin, also have an impact [42,43]. Some investigations reported the presence of cyanobacteria and their MCs in irrigation water sources such as groundwater, natural reservoirs, lakes, and rivers [43,44,45,46,47]. 

Several studies found harmful cyanobacteria and important MC concentrations in irrigation water (Table 1). Quantities of up to 2100 µg/L of extracellular MCs and 3240 µg/g of intracellular MCs can be produced by these species (Table 1). These levels are high in irrigation water and can constitute a health risk, because they exceed the threshold set by WHO recommendations, namely, 1 µg/L of MCs in the lifetime of water and 12 µg/L in the short term of water in drinking water, and 24 µg/L in recreational water, in the absence of regulations on irrigation water quality [48]. Furthermore, several studies showed that both cyanobacteria and their MCs pose a severe problem in irrigation water because of the substantial risk to human health related to their presence in these waters; these water sources may be unsuitable for use as plant irrigation [49]. No study has specifically collected data on the occurrence of MCs in terms of concentrations, variants, and species in irrigation water.

## 4. Phytotoxicity of MC on Crops in Hydroponic Systems

MCs are reported to have several potent impacts on plant growth, physiology, and the yield and quality of fruits (Table 2). Studies have shown that the exposure of hydroponically grown plants to MCs can produce a significant inhibition of root growth of *Oryza sativa* and *Fragaria vulgaris*, and a reduction in plant weight, length, and surface area. We can say that in this type of culture, the roots are in direct contact with the toxin in the absence of soil, which constitutes a barrier against MCs [63,64]. *Medicago sativa* germination, total fresh weight, root, and shoot length were all reduced after 14-day exposure to 5–1000 µg/L of MCs [65]. According to [66], the leaf weight of *Spinacia oleracea* decreases considerably after 21 days of MC exposure (50 and 100 µg/L). Numerous investigations have demonstrated that *Oryza sativa* roots, stems, and leaves exhibit increased biomass, surface area, and dry weight when exposed to low concentrations of MCs [67,68,69]. Exposure to increased concentrations of MC for 7 days had negative effects on the growth of rice plants, reflected in a decrease in the dry weight of roots, stems, and leaves, in addition to the inhibition of photosynthesis [70]. The proportion of fruit set, the number of grains per panicle, and grain weight per panicle all decreased [67]. On the other hand, the early exposure of rice seedlings to MCs (100–1000 µg/L for 7 days) reduced the number of filled grains per panicle, the germination rate, panicle weight, and the contents of soluble proteins, sugars, and starch in the grains [67] (Table 2). 

Concerning oxidative stress, exposure to MCs upstream increased the activity of oxidative enzymes such as superoxide dismutase (SOD), catalase (CAT), peroxidase (POD), polyphenoloxydase (PPO), and ascorbate peroxidase (APX) in *Fragaria vulgaris* plants [62]. 

Several studies have determined that exposure to *M. aeruginosa* extracts containing MCs increases the content of oxidative stress bioindicators such as H_2_O_2_, O_2_, and MDA in *Oryza sativa* L. seedlings [70]. Contact of MCs and plants in hydroponic systems enhances the bioavailability and transport of MCs to various plant organs and induces yield loss. The existence of MCs in hydroponic systems is a fundamental problem that must be carefully addressed to reduce any potential negative effects on plants’ health and yield, since hydroponic systems are gaining more attention, as an alternative to conventional agricultural systems.

**Table 2:** The impact of MCs on the morphological and physiological parameters of various agricultural species in hydroponic systems.

Plant Species	Toxin	Growth Stage	Exposure Time (Days)	Applied Concentration (μg/L)	Phytotoxicity on Plant	References
*Spinacia oleracea*	MC-LR	Plants	21	50	(-) leaf FW	[66]
*Cucumis sativus*	MCt	Seedlings	7	5	(+) H_2_O_2_, O_2_-, MDA	[71]
		Seedlings/flowering /fruiting	7	1–1000	(-) stem diameter, plant height, leaf area, root DW, leaf number, shoot DW, yield	[72]
*Lactuca sativa*	MC-LR (P)	Plants	10	1–100	(-) leaf biomass, root biomass	[73]
		Seedlings	14	5–1000	(-) root length, total FW, shoot length	[74]
*Vicia faba*	MCt	Seedlings	48	10–100	(-) shoot DW, root DW, nodule number, and DW	[75]
*Oryza sativa*	MCt	Seedlings	7	5–500	(-) plant height, shoot DW, root length, root DW; (+) membrane permeability	[76]
*Oryza sativa*	MCt	Seedlings	7	1-3000	(+) root biomass, (-) stem biomass, leaf biomass, grains per panicle, grain weight per panicle, root biomass, setting percentage	[68]
*Oryza sativa*	MCt	Seedlings	7	1–100	(+) root surface area, shoot height; (-) shoot height, root surface area	[67]
*Oryza sativa*	MCt	Seedlings	7	1–3000	(-) stem dry weight, leaf DW, net photosynthetic rate, root DW	[77]
*Oryza sativa*	MCt	Seedlings	7	5–10	(+) H_2_O_2_, O_2_-, MDA; (-) RGR	[71]
*Oryza sativa*	MCt	Seedlings	7	10–1000	(-) root surface area, plant height, filled grains per panicle, panicle weight, seed setting rate, soluble protein, sugar, and starch in the grain	[78]
*Oryza sativa*	MCt	Booting	7	10–1000		
*Oryza sativa*	MCt	Filling	7	10–1000		
*Oryza sativa*	MCt	Seedlings	21	10	(+) Phosphorus content in shoots and roots, root and shoot DW; (-) root DW	[69]
*Oryza sativa*	MCt	Seedlings	30	5–500	(-) root length, root surface area, root dry weight, surface area and volume, root volume, lateral root number, crown root number	[71]

(-): increase; (+): decrease; P: pure MC; DW: dry weight; FW: fresh weight; MCt: total MCs; MDA: malondialdehyde; RGR: relative growth rate; H_2_O_2_: hydrogen peroxide; O_2_-: superoxide anion.

## 5. Bioaccumulation of MCs in Tissues of Plants in Hydroponic Systems

To date, information on the bioaccumulation of MCs by plants in hydroponic crops irrigated with water contaminated with cyanobacterial cells and toxins remains limited. This raises concerns about the mechanisms of bioaccumulation and the health consequences associated with this phenomenon. Nevertheless, studies have shown that MCs can accumulate in terrestrial plants, especially food crops, making them vulnerable to toxin bioaccumulation [79,80]. In hydroponic crops, found that *Lactuca sativa* exposed to concentrations of 5 to 2000 µg/L of MC-LR for 14 days absorbed the toxin at a high rate (700–1400 µg MC-LR/kg FW) [74], surpassing the WHO recommended daily dose of 0.04 µg kg^−1^ body weight [81] (Table 3). Similarly, irrigation of the *Oryza sativa* plant in three hydroponic cultures with water containing different concentrations of MCs showed bioaccumulation of the order of 30 µg/kg in leaves [78], 112,000 µg/kg in leaves [65], and 275 µg/kg in roots [77] (Table 3). Similarly, [72] showed that MCs bioaccumulated in *Cucumis sativus* L. fruits following exposure to concentrations ranging from 1 to 1000 μg/L, and in *Oryza sativa* roots, stem, and leaves following exposure to concentrations ranging from 1 to 3000 μg/L. Indeed, during the process of bioaccumulation, the levels of MCs can potentially decrease to the point of falling below the detection threshold. This phenomenon could be related to the gradual chemical changes that MCs undergo, either as a result of their binding to intrinsic biomolecules or because of detoxification by conjugation with GSH, catalyzed by GST [82]. Alternatively, the diminished detectability of MCs could be attributed to their increased dilution, owing to factors like larger fruit size, greater fruit numbers on plants, and water content during fruit growth and ripening [83]. Plants tend to be affected by several parameters that influence the accumulation of MCs, such as exposure duration (which is most noticeable when it is less than 15 days), concentration, the studied plants, and the particular organ (roots are frequently showing higher absorption rates) (Table 3). 

## 6. Transfer and Fate of MCs in the Terrestrial Food Chain

A crucial route for MC transfer to plants in hydroponic systems is the use of cyanobacterial cell- and MC-contaminated water sources [80]. Under natural circumstances, MCs retain their chemical stability due to their cyclic heptapeptide structure. They are resistant to chemical destruction in extremely acidic and alkaline settings [84], elevated temperatures [85], and photolysis when exposed to natural sunshine [86]. These properties increase MCs’ persistence and availability to plants in irrigation water [87,88]. Moreover, the decomposition of cyanobacterial cells and MCs, introduced into the hydroponic systems, is delayed, leading to the prolonged release of MCs within the substrate and consequently a long-term persistence [89]. In this crop system, roots come into direct contact with MCs, where their degradation is relatively low, enhancing the transfer rate [90]. 

Several studies have been conducted in recent decades on the interactions of MCs with a variety of aquatic vertebrate and invertebrate organisms [91,92,93,94,95]. MC bioaccumulation was investigated in insect chironomid larvae that meet contaminated water surfaces [96]. This study suggests that invertebrates may be essential to the MC translocation to environments with higher trophic levels. A large amount of MCs, however, is delivered to the spleens of ducks, moles, turtles, aquatic birds, and newly emerging aquatic insects. These species may act as carriers, allowing MCs to move from water to terrestrial food chains [97,98,99]. Likewise, exposure through the consumption of imagos and subimagos of *Hexagenia limbata* showed a transfer of MCs to brown bats (*Myotis lucifugus*) [16]. In addition, significant concentrations of MCs were detected in spiders and birds consuming emergent aquatic insects in riparian areas [15]. As a result, land animals may consume meals based on plants irrigated with these MCs or come into direct contact with MC-contaminated water. It has been suggested that MC transmission via ingestion is one of the most serious threats to the terrestrial food chain. There have been few studies on MC transmission from aquatic ecosystems to terrestrial food chains. This scarcity highlights the need for additional research to properly understand the related potential health and ecological implications of MC exposure in terrestrial ecosystems.

## 7. Effects of MCs on Domestic/Wild/Aquatic Animals and Human Health Risks

The presence of toxic cyanobacterial blooms endangers the survival and well-being of several aquatic organisms exposed to MCs, including fish, turtles, ducks, and other species [93]. Studies have demonstrated that MCs can cause a variety of abnormalities and health problems in various fish species, such as coiled bodies and tails, enlarged livers, hepatic hemorrhage, reduced yolk absorption, vacuolar dystrophy, cardiac problems, and disturbances to both organ functions and developmental processes [100,101], in addition to an increase in glutathione –S- transferase (GST) activity in freshwater snails [102,103]. At the cell level, MCs disrupt protein phosphatase activities. The MeAsp residues first prevent PP1 and PP2A from functioning. The cysteine and serine residues of PP1 and PP2A are then covalently bound by the Mdha group of MCs, which permanently inhibits these phosphatases and causes liver damage [104]. MCs have exhibited a noteworthy effect on animals, and damaged the hypothalamic–pituitary system in men by altering mRNA expression and blood hormone levels [105]. Furthermore, studies have shown that MCs can promote tumors, cause acute lung inflammation in rats, alter the structure and function of the testes, induce normocytic anemia, disturb the bone marrow and the immune system, and cause cardiotoxicity in animals [106,107,108,109]. In addition, multiple investigations have shown deadly MC poisonings in domestic and wildland animals [110,111], such as cattle [112], cows [20], sheep [113], heifers [21], dogs [23,114], white rhinos [115], and wild deer [20], zebras, blue wildebeests, and impalas [24]. 

Microcystin-contaminated irrigation water is a common route of MC transfer to agricultural land. As a result, MCs can accumulate within food crops and induce negative effects on human health [16]. After entering the bloodstream, MCs can cause serious organ damage via several biological processes. MCs selectively disturb organs like the liver, kidney, gut, heart, and gonads [19]. Furthermore, MCs might cause an imbalance in cellular oxidation and increase MDA levels in cells, leading to lipid and peroxidation and compromising cell defense ability. This process causes abnormal cell apoptosis, disruption, mitochondrial dysfunction and cytoskeleton endoplasmic reticulum, chronic poisoning, DNA damage, and the emergence of symptoms like nausea, dizziness, vomiting, and other side effects [116,117]. MCs can migrate to vital biological organs such as the heart, brain, and lungs, causing long-term damage in humans [17,18]. Furthermore, MCs have been proven to pass the blood–brain barrier, resulting in unforeseen long-term side effects in humans [118,119].

MC exposure causes glycogen loss, liver cancer, and hepatocyte necrosis [120]. Extensive liver injury can impair bile pigment production, eventually leading to internal bleeding or death from hemorrhage. Hemorrhagic shock has the potential to be fatal to the liver [121]. Similarly, MC exposure alters microRNA expression in liver cells, resulting in severe liver damage and an increase in the formation of liver tumors [122]. Chronic exposure has deleterious consequences, as evidenced by a decrease in human kidney cell viability and the induction of genes involved in apoptosis [123,124]. Cyanobacterial blooms and their associated toxins are widespread in irrigation water sources around the world. Several studies showed their ability to affect biological processes by disturbing organisms’ normal physiology and biochemistry. Despite this, research on the toxicity of MCs and the severe health threats they bring to humans and terrestrial species is still minimal. As a result, more research and tests are needed to fill these gaps and completely assess the MC’s associated impacts on the trophic chain. 

## 8. Conceptual Diagram of MC Fate in a Terrestrial Food Chain

**Figure 2:**
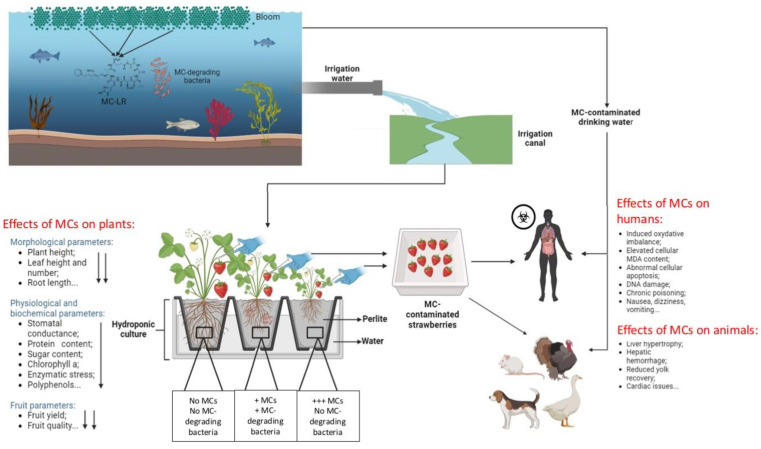
Diagram showing the bioaccumulation, transfer, and effect of MCs in the terrestrial ecosystem.

Eutrophication is a process related to an excessive enrichment of waterbodies with minerals and nutrients, often because of land runoff, leading to an excessive growth of toxic microalgae. This phenomenon can lead to the release of MCs produced by different cyanobacteria species. The use of contaminated water for irrigation purposes leads to possible MC bioaccumulation in various organisms of the terrestrial food web, notably plants (direct contact with MCs) and other terrestrial animals and humans (indirect transfer of MCs). Research has shown that MCs can accumulate in various organs of terrestrial organisms, leading to deleterious impacts on animal and human health. The bioaccumulation of MCs in the food web has been rarely studied in various locations, highlighting the potential risks associated with MC transfer in the food chain [125,126,127,128] (Figure 2).

## 9. Depuration through the Conjugation of MCs

The ADDA group on MC-LR is important for the binding of the toxin to its target enzyme, protein phosphatases 1 and 2A [75]. Moreover, the Mdha group in MCs can subsequently covalently bind to cysteine in a protein phosphatase enzyme [19]. MCs permanently block the active site and destroy the functionality of the protein phosphatase enzyme. In response to exposure to MCs and their negative effects, several organisms have developed detoxification processes for MCs. However, several enzymes are involved in the process of eliminating toxic products [129,130]. 

Studies on the detoxification mechanism of MCs in plant tissues are limited. After the absorption of MCs by the plant, a certain quantity will be conjugated by nonenzymatic glutathione (GSH). A second quantity will be enzymatically conjugated into GSH via the glutathione S-transferase system. Part of the remaining MC-LR binds to phosphatases and possibly other cellular proteins. Finally, the remaining MC-LR could be taken by chloroplasts, in which three main pathways have been postulated, a non-enzymatic pathway by binding to the GSH, enzymatic conjugation to GSH via the GST system, and reactions with proteins or structures of the photosynthetic apparatus of the chloroplast. To remove conjugates from glutathione, plants transfer these conjugates into the vacuole through multi-drug resistance proteins (MRPs), which are part of the ABC family of conveyors, for the temporary storage and subsequent processing of GSH conjugates [75].

**Figure 3:**
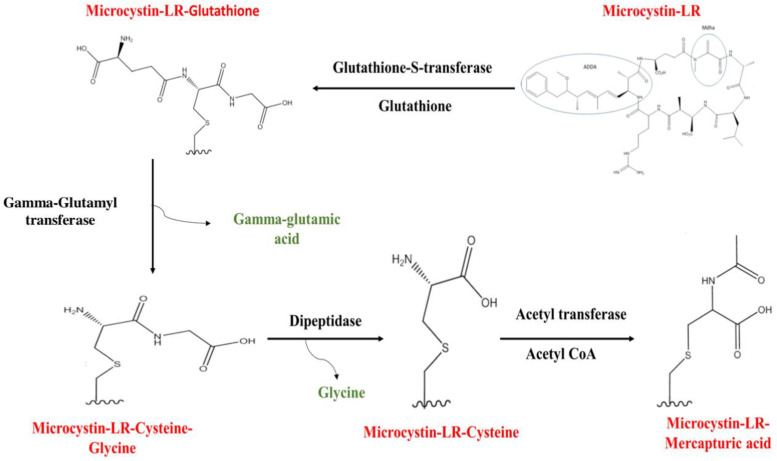
Glutathione metabolic pathway for microcystin-LR [132].

In animals, when the toxin arrives in the cell, oxygen-containing groups are attached to the toxins, and phase I enzymes, notably cytochrome P-450, initiate oxidative reactions. Phase II enzymes, such as glutathione-S-transferase (GST), intervene to modify the toxin and form glutathione conjugates. Glutathione (GSH) is commonly used in phase II biotransformations. The formation of the glutathione conjugate by the phase II enzyme, glutathione-S-transferase (GST), is one of the most common types of toxin modification [129]. This reaction occurs between a nucleophilic center of the toxin (Mdha group) and the sulfhydryl group of reduced glutathione (GSH) [129]. When a toxin conjugates with GSH, the enzyme γ-glutamyl transferase cleaves the γ-glutamic acid group of the GSH molecule to produce the intermediate γ-glutamylcysteine [129,131]. The glycine of this γ-glutamylcysteine intermediate is subsequently cleaved by a dipeptidase to create a cysteine-conjugated product, which is ultimately oxidized to yield the mercapturic acid metabolite. While other metabolic pathway conjugates may be excreted from cells, this mercapturic acid derivative is easily eliminated in the urine [129] (Figure 3). 

## 10. Approaching the Bacterial Enzymatic Biodegradation of MCs

The quality of irrigation water must be assessed to prevent short- or long-term potential negative effects of MCs. To protect household, agricultural, and recreational applications, water sources and supply need to be monitored for the possible presence of hazardous cyanobacteria and MCs. When treating toxin-contaminated water, the physical and chemical properties of the toxin, its nature, and the development and proliferation patterns of cyanobacteria must all be taken into account [133]. There have also been attempts to remove MCs from irrigation-grade water using treatment techniques such as ozonation and chlorination [134]. Several studies stated that standard water treatment techniques can be costly to maintain, ineffective in getting rid of or degrading MCs, and potentially hazardous by their byproducts. Therefore, it is imperative to investigate a treatment strategy that is economical and efficient while also not endangering the environment or producing unfavorable byproducts after treatment. Several studies focused on using bacteria as a biological treatment approach to break down and eliminate MCs from water. MC breakdown has been seen in a variety of different taxa, including *Sphingomonas*, *Arthrobacter*, *Acinetobacter*, *Novosphingobium*, *Bacillus*, *Paucibacter*, *Pseudomonas*, *Stenotrophomonas*, and *Sphingopyxis* [11,135,136]. The rate of degradation varies amongst different strains; *Bacillus* sp. AMRI-03, for instance, took five days to fully break down MC-RR [137]. Within 72 h, *Arthrobacter* spp. F10, F7, C6, R1, R4, R6, and R9 were able to eradicate MC-LR [138]. In 24 days, MC-LR was eradicated by *Pseudomonas aeruginosa* DMXS and *Novosphingobium* sp. KKU-25s [139]. Furthermore, MC-LR, MC-LF, and MC-RR were eliminated by *Stenotrophomonas maltophilia* 4B4 in 10, 12, and 14 days, respectively [140]. The *Sphingopyxis bacterium* YF1 is the fastest; it was reported to completely degrade MC-LR within 120 min [141]. Based on the existing evidence, bacterial strains globally widespread in several habitats possess a capacity for chemical compound breakdown and may play a major role in natural processes [142]. Furthermore, it was shown that a mixture of different bacterial strains may break down MC-LR. For example, the complete degradation of MC-LR was demonstrated by a combination of ten isolates (*Aeromonas* sp., *Acinetobacter* sp., *Novosphingobium* sp., *Pseudomonas* sp., *Ochrobactrum* sp., *Sphingomonas* sp., *Rhodococcus* sp., *Sphingopyxis* sp., *Stenotrophomonas* sp., and *Steroidobacter* sp.), and another combination of seven isolates (*Hyphomicrobium aestuarii*, *Acinetobacter* sp., *Rhizobium* sp., *Pseudoxanthomonas* sp., *Sphingomonas* sp., *Sphingobium* sp., and *Steroidobacter* sp.) [142]. A naturally occurring group of bacteria collected from the mucilage of *M. aeruginosa* colonies during their flowering stage, comprising *Agrobacterium* sp., *Brevundimonas* sp., *Bosea* sp., *Rasbo* sp., *Hyphomicrobium* sp., *Rhizobium* sp., *Roseomonas* sp., *Rhodococcus* sp., *Mesorhizobium* sp., *Nitrosococcus* sp., *Sphingomonas* sp., and *Sandaracinobacter* sp., effectively decomposed MC-LR [142,143]. 

Several studies focusing on genotypic analysis have identified specific gene clusters, including *mlrA*, *mlrB*, *mlrC*, and *mlrD*, in certain bacterial groups. These gene clusters encode enzymes such as MlrA, MlrB, MlrC, and MlrD, which are involved in the degradation of MCs [141,142].

The degradation process begins with the cleavage of the Adda-Arg peptide bond of MC-LR by the enzyme MlrA, which is encoded by the *mlrA* gene and produces linearized MC-LR. The linearized MC-LR is subsequently converted into a tetrapeptide by the enzyme MlrB, which is generated by the *mlrB* gene. This process involves hydrolyzing the Ala-Leu link. Furthermore, the tetrapeptide is broken down into smaller peptides and amino acids by the enzyme MlrC, which is expressed by the *mlrC* gene. It is believed that the *mlrD* gene facilitates the transmission of MCs and their breakdown products [144]. 

**Figure 4:**
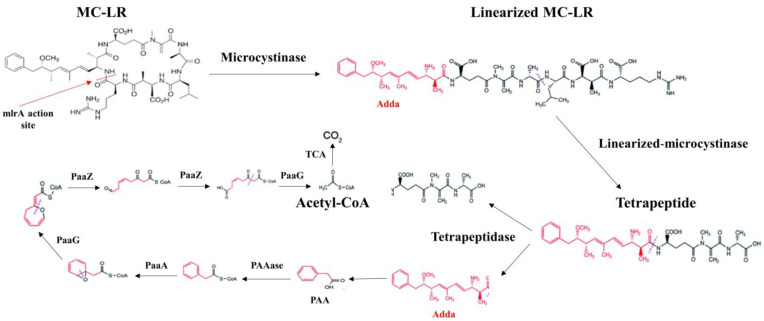
Biodegradation pathway of MC-LR by *Sphingopyxis* sp. YF1. A potential connection was found between the *mlrABCD* gene cluster and the *paa* gene clusters involved in the degradation of phenylacetic acid (PAA). The process of degradation proceeded as follows: sequentially, MC-LR was broken down into linearized MC-LR, Adda, and tetrapeptide. After that, Adda was broken down into PAA, and PAAase was then changed into PAA-CoA. PAA-CoA was further broken down to acetyl coenzyme A (acetyl-CoA) by PaaA homologs PaaG and PaaZ. Ultimately, acetyl-CoA was entirely transformed into CO_2_ by the tricarboxylic acid (TCA) cycle. Based on available data, it is highly probable that PAA is the subsequent metabolic product resulting from Adda’s conversion to PAA [141].

As a result, breakdown routes may differ between MC genotypes and bacterial strains [144]. In addition to well-known MC-LR degradation products (tetrapeptide, adda, and linearized MC-LR), studies have identified eight other degradation intermediaries: three tripeptides (ADA-Glu-MDHA, GLU-MDHA-ALA, and LEU-MEASP-ARG), three dipeptides (GLU-MDHA, MDHA-ALA, and MESP-ARG), and two amino acids (Leu and ARG) for the MC-LR degradation pathway use of *Sphingopyxis* sp. [141,145]. These biodegradation pathways are important for developing strategies to degrade MCs in contaminated environments (Figure 4).

## 11. Conclusions and Research Requirements

In recent years, research into cyanobacteria and their toxins has developed very rapidly around the world. However, it is clear from the data synthesized and presented above that the distribution of MCs in irrigation water, their transfer and bioaccumulation in hydroponically grown plants and animals, their phytotoxicity and effects on animals and humans, and the ways in which MCs are eliminated are still poorly understood. This research aims to synthesize existing knowledge on the fate and effects of MCs that contaminate the irrigation water of hydroponic crops and to propose methods for eliminating these toxic substances in order to mitigate the associated risks. Many studies have shown that MCs released into irrigation water can migrate to plant roots, then to leaves, to accumulate in edible parts like fruit. This bioaccumulation of MCs can negatively affect plant morphological and physiological parameters and fruit yield and quality. The assessment of the phytotoxicity and bioaccumulation of MCs in agricultural plants, particularly in hydroponic crops, highlighting the potential risks to plants associated with the presence of MCs in irrigation water, requires further and more detailed research. In addition, MCs can be transferred and bioaccumulated in various animal and human tissues, leading to adverse effects in animals and humans. This shows that MCs can enter the terrestrial food chain through the ingestion of edible plant parts or drinking water. However, the transfer of MCs to terrestrial animals through the direct consumption of edible agricultural products, particularly those grown hydroponically, has not been considered a major concern, despite its potential impact on animal health and the food chain in general. To this end, the efforts of researchers concerned with MCs are focusing on approaches to eliminating MCs, either by eliminating cyanobacteria and MCs from water or by investigating MCs’ detoxification mechanisms in animal and plant cells affected by MCs. 

This comprehensive review addresses many critical aspects of MCs, including their distribution in irrigation water, their transfer and bioaccumulation in hydroponic food plants, their transfer to terrestrial animals, and the impact they have on flora and fauna, including humans; this research highlights the ubiquitous nature of these toxins in terrestrial ecosystems. Understanding these multifaceted interactions provides a holistic perspective essential to the development of effective strategies to mitigate the negative consequences of MCs on hydroponic agricultural products, animal welfare, and human well-being. Such an integrated approach is essential to promote informed decision-making and develop sustainable solutions to preserve the quality of irrigation water and hydroponic crops, and to protect ecosystems and human health from the threats posed by MCs. 

## Figures and Tables

**Table 1:** Occurrence of MC congeners in freshwater destined for irrigation around the world.

Lakes/Reservoirs	Location	Cyanobacteria	Extracellular MCs (µg/L)	Intracellular MCs	Congeners	References
Hakanoa Lake	North Island, New Zealand	*Microcystis aeruginosa*; *Anabaena* cf. *smithii*	2100	ND	MC-RR, -LR, -FR, -WR, -LY, -AR, -LA, and YR	[50]
Köyliönjärvi Lake	southwest Finland	*Microcystis* spp. (*M. wesenbergii*, *M. botrys* and *M. aeruginosa*); *Dolichospermumsp*., and *Aphanizomenon* sp.; *Planktothrix* sp.; *Aulacoseira ambigua and Cyclotella* spp.; *Planktolyngbya limnetica*;	0.12–0.28	0.97–2.4 µg/L	MC-RR, -LR, -YR, and -RR	[46]
Groundwater wells	Asir region, Saudi Arabia	*Oscillatoria limnetica*	0.3–1.8	336 µg/g	MC-RR, -LR, and -YR	[51]
Karla Reservoir	Central Greece	*Planktothrix* cf. *agardhii*; *Anabaena* sp.	1.5–33	ND	MC-LR, and -RR	[52]
Dianchi Lake; Xingyun Lake	Yunnan province, China	ND	0.117–46.7	0.062–514.6 µg/L	MC-RR, -LR, and -YR	[53]
Dashahe Reservoir	Jiangmen, Guangdong province, China		0.016–3.1	0.594–450.7 µg/L		
Karla Reservoir	Central Greece	ND	3.8	ND	MCt	[54]
Nile river	Egypt	*Anabaena subcylindrica*; *Nostoc spongiaeforme*; *Plectonema boryanum*; *Phormidium corium*; *Aulacoseira ambigua and Cyclotella* sp.	ND	1.6–3.66 µg/L	MC-RR, and -YR	[47]
Lalla Takerkoust reservoir	Marrakech, Morocco	*Microcystis aeruginosa* Kütz	60	3240 µg/g	MCt	[43]
Taihu Lake	Suzhou, China	*Microcystis*	4.14	17.57 µg/L	MC-RR, -LR, and -YR	[55]
Occhito reservoir	Italy	ND	0.18 µg/L	ND	MC-LR, -RR, -LA, -YR, -LY, -LF, and -LW	[56]
Karla Reservoir	Central Greece	*Microcystis aeruginosa*	1.43–2.03	ND	MC-LR, and -RR	[57]
Sources of irrigation water	Egypt	*Oscillatoria limnetica* and *Microcystis aeruginosa*	45.04–600	58,000–87,000 µg/L	MC-LR, and -RR	[58]
Beira Lake	Sri Lanka	*Microcystis aeruginosa*	180	340 µg/g	MC-LR	[59]
Irrigation heads; Irrigation intakes; Epilimnion of surface water sources	Missouri and Kansas, USA	ND	8.53–8.65	ND	ND	[46]
Mansour Eddahbi Reservoir	South of Ouarzazate city, Morocco	*Microcystis aeruginosa* Kütz; *Pseudanabaena papillaterminata* Kuk; and *Oscillatoria* sp.	ND	64.4 μg/g	MC, -LR, -RR, -YR, -FR, and -WR	[60]
Lalla Takerkoust reservoir	Central regions of Morocco	*Microcystis aeruginosa*	ND	2.2–944 µg/g	MC-LR	[61]
Reservoirs of the river Segura	Murcia, SE Spain	*Aphanizomenon flos-aquae* and *Microcystis aeruginosa*	0.067–1.586	ND	MC-RR, -LR, and -YR	[62]

ND: Not determined; MCt: Total MCs.

**Table 3:** A view of MC bioaccumulation in hydroponically irrigated plants.

Plants Species	Applied Concentration (μg/L)	Organs	Concentration Accumulated (µg/kg)	Exposure Time (Days)	EDIAdu/EDIchi (μg/kg)	BFA of MCs	Reference
*Lactuca sativa*	5–2000 MC-LR (P)	Leaves	700–1400 MC-LR FW	14	0.43–0.86/0.28–0.56	140/0.7	[74]
*Cucumis sativus* L.	1–1000 CE FW MCt	Fruit	2.87–29.64 MCt FW	7	0.001–0.018/0.001–0.011	0.29	[72]
*Oryza sativa*	5–500 CE FW MCt	Roots	≈7000–35,000 MCt FW	30	-	1400/70	[76]
		Stems	≈10,000–38,000 MCt FW		-	2000/76	
		Leaves	≈20,000–112,000 MCt FW		-	4000/224	
*Oryza sativa*	1–3000 CE FW MCt	Roots	≈20–275 MCt FW	7	-	20/0.091	[78]
		Stems	≈18–30 MCt FW		-	18/0.01	
		Leaves	≈22–230 MCt FW		-	22/0.076	
*Oryza sativa*	9.79 CE FW MCt	Roots	≈1.22 MCt FW	21	-	0.12	[69]
		Leaves	≈0.98 MCt FW		-	0.1	
		Leaves	1.55–6.59 MCt FW		0.001/0.0005	1.55–0.32	
		Fruit	1.04 MCt FW			0–0.052	

Not determined (ND); Crude extract (CE); Pure toxin (P); Estimated daily intake (EDI); Estimated daily intake for adult (EDI_Adu_); Estimated daily intake for children (EDI_Chi_); Bioaccumulation factor (BFA); Total MCs (MCt); Fresh weight (FW).

## Data Availability

No new data were created.
